# Efficacy of coenzyme Q10 in patients with cardiac failure: a meta-analysis of clinical trials

**DOI:** 10.1186/s12872-017-0628-9

**Published:** 2017-07-24

**Authors:** Li Lei, Yan Liu

**Affiliations:** 1 Department of Cardiology, Yulin Traditional Chinese Medicine Hospital, Yulin, 719000 China; 2 First Department of Cardiology, Yulin Second Hospital, South Wenhua Road, Yulin, 719000 China

**Keywords:** Coenzyme Q10, Cardiac failure, Exercise capacity, Placebo

## Abstract

**Background:**

The therapeutic efficacy of coenzyme Q10 on patients with cardiac failure remains controversial. We pooled previous clinical studies to re-evaluate the efficacy of coenzyme Q10 in patients with cardiac failure.

**Methods:**

We searched PubMed, Cochrane Library, EMBASE, and Clinical Trials.gov databases for controlled trials. The endpoints were death, left heart ejection fraction, exercise capacity, and New York Heart Association (NYHA) cardiac function classification after treatment. The pooled risk ratios (RRs) and standardized mean difference (SMD) were used to assess the efficacy of coenzyme Q10.

**Results:**

A total of 14 RCTs with 2149 patients met the inclusion criteria and were included in the analysis. Coenzyme Q10 decreased the mortality compared with placebo (RR = 0.69; 95% CI = 0.50–0.95; *P* = 0.02; *I* ^*2*^ = 0%). A greater improvement in exercise capacity was established in patients who used coenzyme Q10 than in those who used placebo (SMD = 0.62; 95% CI = 0.02–0.30; *P* = 0.04; *I* ^*2*^ = 54%). No significant difference was observed in the endpoints of left heart ejection fraction between patients who received coenzyme Q10 and the patients in whom placebo was administered (SMD = 0.62; 95% CI = 0.02–1.12; *P* = 0.04; *I* ^*2*^ = 75%). The two types of treatment resulted in obtaining similar NYHA classification results (SMD = −0.70; 95% CI = −1.92–0.51; *P* = 0.26; *I* ^*2*^ = 89%).

**Conclusion:**

Patients with heart failure who used coenzyme Q10 had lower mortality and a higher exercise capacity improvement than the placebo-treated patients with heart failure. No significant differences between the efficacy of the administration of coenzyme Q10 and placebo in the endpoints of left heart ejection fraction and NYHA classification were observed.

**Electronic supplementary material:**

The online version of this article (doi:10.1186/s12872-017-0628-9) contains supplementary material, which is available to authorized users.

## Background

Heart failure is a complex clinical syndrome that results in an inadequate cardiac output and reduced ejection capacity due to serious structural or functional abnormalities of the heart. Millions of people are diagnosed with heart failure worldwide every year [1], which is the most frequent cause of hospitalization and disability [2, 3]. Although there have been major improvements in the pharmacological management of heart failure, death rates continue to exceed 10% per year, reaching between 20% to 50% in severe cases [4]. Supplementary oral administration of coenzyme Q10 has been found to increase coenzyme Q10 levels in plasma, platelets, and white blood cells [5]. Studies also evidenced that the concentration of coenzyme Q10 in the plasma of patients with heart failure is an independent predictor of heart failure death [6]. However, most of the reported clinical trials have been performed on a limited sample size. For example, Mortensen [7] reported that coenzyme Q10 decreased the mortality and improved the left heart ejection fraction compared with the placebo used, whereas no significant difference between the endpoints of mortality and left heart ejection fraction were found by Khatta [8]. We pooled previous studies to evaluate the efficacy of coenzyme Q10 in heart failure treatment to provide a data-driven concept for clinical practice.

## Methods

### Data sources and searches

We searched PubMed, Cochrane Library, EMBASE, and Clinical Trials.gov databases from database inception until December 25 2016, using the keywords “coenzyme Q10”, “ubiquinone Q10”, “cardiac failure”, and “heart failure”. A sensitive filter for randomized controlled trials was utilized for the search. In addition, references from randomized trials and relevant reviews were hand-searched for additional trials that were not identified in the database search.

### Study selection

The following inclusion criteria were applied: (1) patients with heart failure; (2) randomized controlled trials comparing the efficacy of coenzyme Q10 versus placebo and other drugs; (3) clinical outcomes were reported, such as death, left heart ejection fraction, exercise capacity, NYHA classification, and adverse events; Reviews, meta-analyses, and observational studies were excluded. The meta-analysis was conducted according to the Preferred Reporting Items for Systematic Review and Meta-analysis (PRISMA) guidelines [9].

### Data extraction and quality assessment

Two investigators independently extracted data from the relevant sources. Authors were contacted when data were incomplete or unclear, and conflicts were resolved by discussion. For the cross-over studies, single-phase data were extracted. Baseline demographic characteristics of the patients (sample size, diabetes percent, age, sex, and intervention in the experimental and control group) were collected from the eligible studies. The occurrence rates of the following events were abstracted: death. Mean and standard values of the following events were abstracted: left heart ejection fraction, exercise capacity, and NYHA classification. According to the guidelines of the Cochrane Handbook for Systematic Reviews of Interventions (version 5.0.2, last update September, 2009); the quality of the information accessed in each of the studies was classified as low, unclear, or high by evaluating the following seven components: random sequence generation, allocation concealment, blinding of participants and outcome assessment, incomplete outcome data, selective outcome reporting, and other issues.

### Data analysis

Binary classification variables of the clinical endpoints were measured by using the risk ratio (RR) with 95% confidence intervals (CIs). The continuous variables of the clinical endpoints were determined by using the standardized mean difference (SMD) with 95% CIs. Two-sided *P-*values <0.05 were considered statistically significant. A random-effects model was used to calculate all pooled estimates. Small-study and publication biases were assessed by funnel plot analysis and Egger’s test. Data analysis was conducted using RevMan 5.2 software (Nordic Cochrane Centre, Cochrane Collaboration, 2013), and sensitivity analysis was performed by Stata 11.0 (StataCorp, College Station, TX, USA).

## Results

### Data search results

**Fig. 1:**
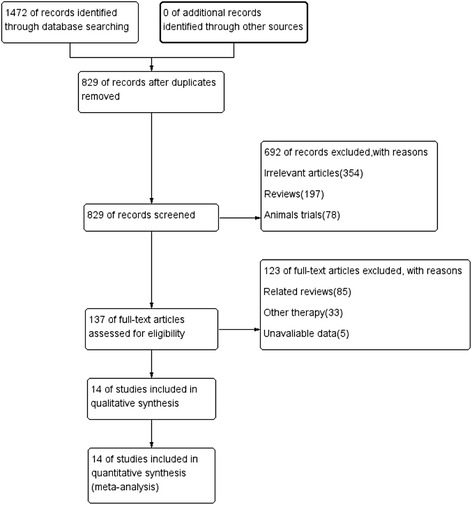
Flow chart showing the progress of data selection

We identified 14 trials [7, 8, 10–21] out of 1472 records, that satisfied our inclusion criteria, as can be seen in the selection procedure depicted in Fig. 1. A total of 1064 patients were randomized to a coenzyme Q10 (treatment) group, and 1085 patients were randomized to a placebo (control) group. The baseline demographic characteristics of the included studies are detailed in Table 1. The quality assessment data are presented in Additional file 1: Fig. S1 and Fig. S2. All clinical trials included in our study were characterized by a low risk of blinding of participants and outcome assessment, and selective outcome reporting. In addition, only one study with complete details of allocation concealment and three studies with unclear risk of incomplete outcome data which we included. In conclusion, the trials included in the present analysis were middle- or high-quality studies.

**Table 1:** Characteristics of the included studies

	Sample size(T/C)	Mean Age(T/C)	Intervation		NYHA	LVEF (%)
			T	C		
Alehagen 2015	221/222	59 ± 9	Coenzyme Q10 100 mg, tid	Placebo	II-III	37 ± 7
Belardinelli 2006	23/23	61 ± 10	Coenzyme Q10 100 mg, qd	Placebo	II-IV	22 ± 10
Hofman-Bang 1995	79/79	62 ± 7/61 ± 9	Coenzyme Q10 50 mg, tid	Placebo	II-III	<40
Keogh 2003	19/20	67	Coenzyme Q10 200 mg, qd	Placebo	III-IV	<40
Khatta 2000	28/27	66/67	Coenzyme Q10 50 mg, tid	Placebo	III-IV	NR
Kocharian 2009	17/21	49.8 ± 6.7	Coenzyme Q10 50 mg, tid	Placebo	II-IV	29 ± 11
Morsico 1993	319/322	62 ± 12/62 ± 11	Coenzyme Q10 100 mg, tid	Placebo	II-IV	31 ± 10
Morsico 1994	6/6	60/54	Coenzyme Q10 100 mg, bid	Placebo	II-III	26 ± 6/31 ± 5
Mortensen 2014	202/218	52 ± 9	Coenzyme Q10 33.3 mg, tid	Placebo	I-III	38 ± 16/40 ± 11
Munkholm 1999	11/11	51 ± 13/55 ± 15	Coenzyme Q10 100 mg, bid + atorvastatin	Placebo	II-IV	19 ± 10/26 ± 10
Permanetter 1992	15/10	63 ± 7/62 ± 6	Coenzyme Q10 30 mg, qd	Placebo	II-IV	38 ± 3/36 ± 4
Pourmoghaddas 2014	32/30	55 ± 11	Coenzyme Q10 33 mg, tid	Placebo	NR	26 ± 6
Watson 1999	30/30	78	Coenzyme Q10 100 mg, bid	Placebo	I-III	NR
Zhao 2015	62/66	6 ± 5/7 ± 5	Coenzyme Q10 2 mg/kg/day in 2 or 3 divided doses	Placebo	NR	29 ± 10/33 ± 10

### Results of mortality

**Fig. 2:**
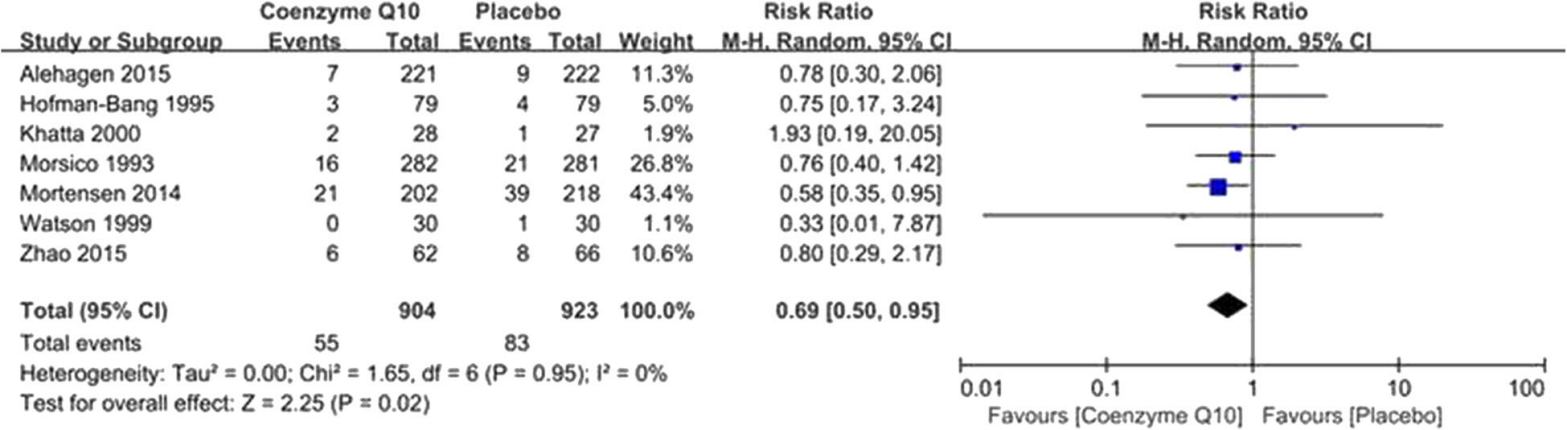
Forest plot of mortality

The analysis of mortality showed that 55 out of 904 patients from the coenzyme Q10 group and 83 out of 923 from the control group died. The mortality was decreased by coenzyme Q10 compared with placebo (RR = 0.69; 95% CI = 0.50–0.95; *P* = 0.02; *I* ^*2*^ = 0%) as shown in Fig. 2.

### Results of left heart ejection fraction

**Fig. 3:**
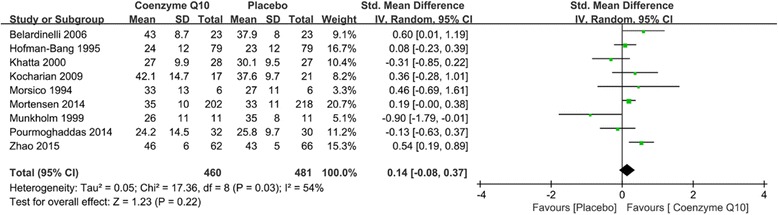
Forest plot of left heart ejection fraction

In our study, nine clinical trials reported the endpoint of left heart ejection fraction, in which 460 patients were assigned to coenzyme Q10 treatment arm and 481 patients were assigned to placebo treatment arm. Patients who used coenzyme Q10 and placebo associated with similar left heart ejection fraction (SMD = 0.14; 95% CI = −0.08–0.37; *P* = 0.22; *I* ^*2*^ = 54%) when the random-effects was used as shown in Fig. 3.

### Results of the exercise capacity test

**Fig. 4:**
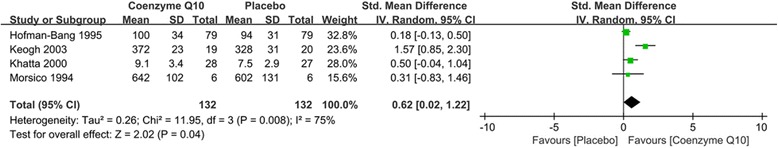
Forest plot of exercise capacity

In our study, four clinical trials reported the endpoint of exercise capacity in which 132 patients received treatment with coenzyme Q10, and 132 patients were treated with placebo. Using the random-effects model, we found that the exercise capacity was more significantly improved in the patients who used coenzyme Q10 (measured as exercise duration or walking distance, or both) than in the patients who used placebo (SMD = 0.62; 95% CI = 0.02–1.12; *P* = 0.04; *I* ^*2*^ = 75%) (Fig. 4).

### Results of NYHA classification

**Fig. 5:**
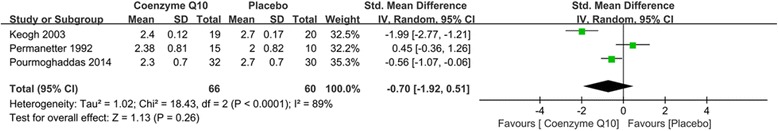
Forest plot of NYHA classification

Three clinical trials reported the endpoints of NYHA classification, in which 66 participants were given therapy with coenzyme Q10, and 60 participants were administered therapy with placebo. The random-effects model used exhibited no significant differences between these two types of treatment (SMD = −0.70; 95% CI = −1.92–0.51; *P* = 0.26; *I* ^*2*^ = 89%) when (Fig. 5).

### Publication bias analysis

**Fig. 6:**
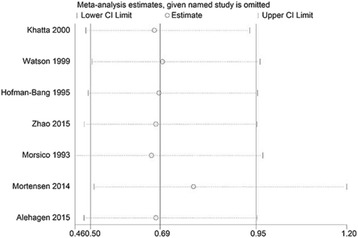
Sensitivity analysis

Egger’s test results showed no significant evidence of publication bias in either endpoint (Table 2). All the Egger’s regression outcomes of these four clinical endpoints had a *P*-value >0.05, which demonstrated that no publication bias was present in our study. As illustrated in Fig. 6, similar results were obtained after excluding each individual study.

**Table 2:** Assessment of Publication Bias

Outcome	Egger regression *P* value
Mortality	0.387
Left heart ejection fraction	0.587
Exercise capacity	0.393
NYHA classification	0.950

## Discussion

Coenzyme Q10 is a ubiquitous fat-soluble compound involved in mitochondrial oxidative phosphorylation. Evidence suggests that this substance with antioxidant properties improves human immunity and overall vitality. Coenzyme Q10 plays an important role in mitochondrial oxidative phosphorylation and the production of adenosine triphosphate [22]. A slight change in the levels of coenzyme Q10 may lead to significant alterations in respiratory rate [23]. In addition, by impediment of the reaction of NO with peroxide, coenzyme Q10 can reduce the overall peripheral resistance and improve the heart ejection function; thus, NO in the endothelial cells increases vascular smooth muscle relaxation, which prevents the occurrence of myocardial ischemia [24–26]. A balance between oxidative and antioxidant activities exists in the patients with heart failure, and the protective function of the antioxidant enzymes in the body is weakened. Oxygen free radicals cause cell damage by activating apoptosis and mitochondrial protein destruction by lipid peroxidation. The lack of coenzyme Q10 in human may increase the rates of heart failure by augmenting the pressure on the heart wall; this leads to an increase in energy expenditure, resulting in an imbalance between energy supply and demand [24]. In a previous study, coenzyme Q10 was founded can exert direct antioxidant effects through enhancing myocardium energy generation by promoting oxidative phosphorylation of the cells. Heart failure patients always have low concentration of coenzyme Q10 and the decrease of coenzyme Q10 with increasing severity of heart failure [10].

In previous studies, coenzyme Q10 reduced the mortality in patients with heart failure, improving heart ejection fraction [27, 28]. Meanwhile, coenzyme Q10 did not significantly improve the NYHA cardiac function in patients with heart failure, which is consistent with the results of a previous examination [29]. In this study, we found that coenzyme Q10 can improve exercise tolerance in patients with heart failure; however, in the investigation conducted by Madmani [30], this improvement was not statistically significant.

Our research is the newest meta-analysis that analyzes the efficacy of coenzyme Q10 in heart failure patients. Compared with the meta-analysis published by Madmani in 2014 [30], we included 14 additional clinical investigations and 2149 more participants than in the previously mentioned meta-analysis. Meanwhile, compare with the meta-analysis conducted by Fotino [29], we analysis the efficacy of coenzyme Q10 in the endpoints of mortality and NYHA classification. Because of the inclusion of different studies, varying results were obtained for the endpoint of exercise capacity. Patients who used coenzyme Q10 extended their exercise capacity to a more considerable extent than patients who received placebo.

Nevertheless, there are some limitations in our meta-analysis. First, the dose of coenzyme Q10 and the duration of treatment were not uniform which might have affected the reliability of our results. Second, several of the trials included were without detailed descriptions of allocation concealment and blinding, which might have led to bias. Third, insufficient clinical information was included on the endpoints of exercise capacity and NYHA classification, which might have caused heterogeneity which we were unable to estimate. In addition, the generally different design and characteristics of each trial might have also caused heterogeneity. Therefore, conducting more rigorous, large-sample, international trials is needed to confirm our results.

## Conclusion

In patients with heart failure, the administration of coenzyme Q10 resulted in lower mortality and improved exercise capacity compared with the effects of placebo treatment. No significant difference was found between coenzyme Q10 and placebo in the endpoints of left heart ejection fraction and NYHA classification.

## Additional file

Additional file 1: Figure S1. Risk of bias graph. Review authors’ judgements about each risk of bias item presented as percentages across all included studies. **Figure. S2.** Risk of bias summary. Review authors’ judgements about each risk of bias item for each included study. (DOCX 613 kb)

## Abbreviations

CIs: confidence intervals
NYHA: New York Heart Association
RRs: pooled risk ratios
SMD: standardized mean difference
