# Supplementary figures and images for: Efficacy of coenzyme Q10 in patients with cardiac failure: a meta-analysis of clinical trials
Source: BMC Cardiovasc Disord. 2017 Jul 24;17:196. doi: 10.1186/s12872-017-0628-9 (PMC5525208; doi:10.1186/s12872-017-0628-9)

**Supplementary Figure 1.** Risk of bias graph


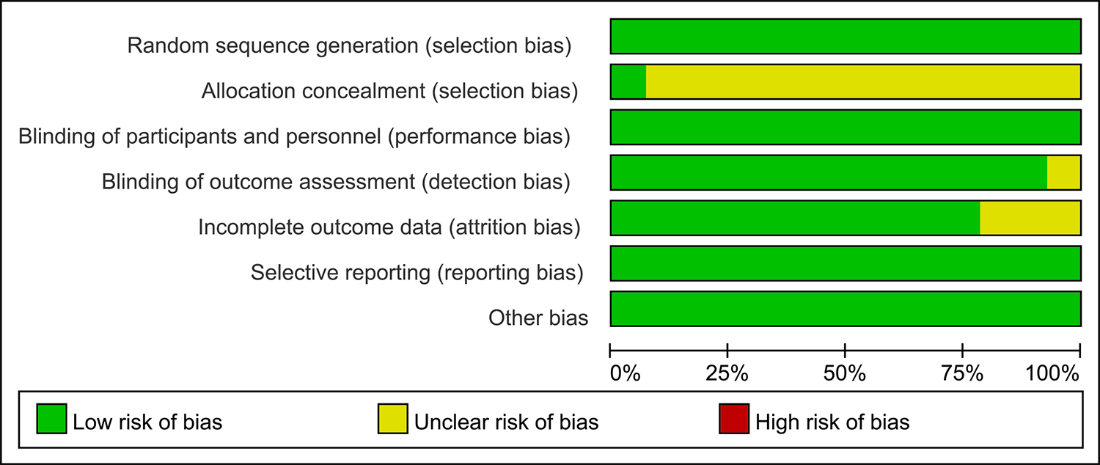


**Supplementary Figure 2.** Risk of bias summary


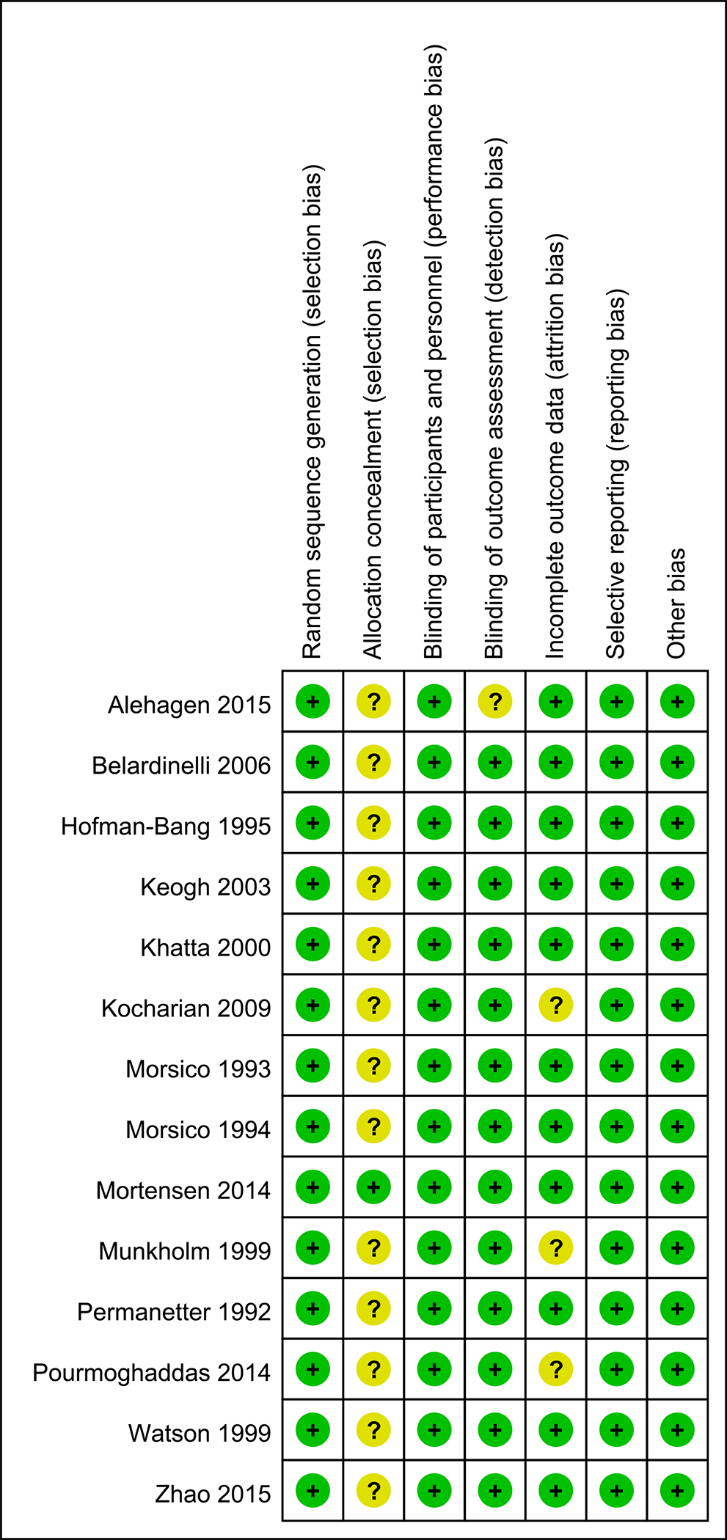

Supplement: Additional file 1: Figure S1. — Risk of bias graph. Review authors’ judgements about each risk of bias item presented as percentages across all included studies. Figure. S2. Risk of bias summary. Review authors’ judgements about each risk of bias item for each included study. (DOCX 613 kb) [file 12872_2017_628_MOESM1_ESM.docx]
